# Chemical Stabilisation of Collagen as a Biomimetic

**DOI:** 10.1100/tsw.2003.13

**Published:** 2003-03-24

**Authors:** R. Gordon Paul, Allen J. Bailey

**Affiliations:** British School of Leather Technology, University College Northampton, Northampton, UK

**Keywords:** collagen, biomimetics, chemical cross-linking, physical cross-linking, biomechanics, calorimetry

## Abstract

Collagen is the most abundant protein in animals and because of its high mechanical strength and good resistance to degradation has been utilized in a wide range of products in industry whilst its low antigenicity has resulted in its widespread use in medicine. Collagen products can be purified from fibres, molecules reconstituted as fibres or from specific recombinant polypeptides with preferred properties. A common feature of all these biomaterials is the need for stable chemical cross-linking to control the mechanical properties and the residence time in the body, and to some extent the immunogenicity of the device. This can be achieved by a number of different cross-linking agents that react with specific amino acid residues on the collagen molecule imparting individual biochemical, thermal and mechanical characteristics to the biomaterial. In this review we have summarised the major techniques for testing these characteristics and the mechanisms involved in the variety of cross-linking reactions to achieve particular properties..

## INTRODUCTION

### The Nature of Collagen Fibres

Collagen is the major protein of the animal body, from the simplest sponges to *Homo sapiens.* The biological diversity of these collagenous structures is unequalled by any other protein, ranging from skin, bone, and tendon to microscopically thin basement membranes. This variety of form can now be explained on the basis of a whole family of genetically distinct collagen molecules that aggregate into different supramolecular structures. Over the past 2 decades, 20 different collagens have been identified[1,2,3]. Despite this diversity, the basic structure of collagen is very simple. The primary sequence is basically a repeating tripeptide $(Gly\text{-}X\text{-}Y)_{100\text{-}400}$ where X is often proline and Y is often hydroxyproline. The chains, therefore, adopt a left-handed polyproline helix and three chains interact to form a right-handed triple helix (Fig. 1). The small side-chain of glycine, a hydrogen atom, allows close contact of the three chains and at the same time stabilises the helix by forming internal hydrogen bonds with the peptide backbone of an adjacent chain. Although the family of collagen molecules form several different types of aggregates, the major collagen of skin, tendon, and bone are fibrous collagens in which the long rod-like molecules (1.5 × 300 nm) are aligned in parallel in a quarter-staggered arrangement. Types I, II, III, and V collagens form fibres and initially possess N- and C-terminal globular domains that are almost completely removed prior to extracellular aggregation of the molecules into the quarter-staggered fibril. The residual globular domains of about 15 to 20 amino acids do not possess the triple helical structure because of their different amino acid sequence and are essential in the enzymic formation of the natural cross-links stabilising the fibril[4].

The chemical cross-linking of collagen has a long and illustrious history in the stabilisation of animal hides to provide leather with specialised properties (summarised in an excellent review by Covington[5]), primarily increased resistance against thermal and biological degradation. However, collagen is now being extensively used for medical and cosmetic application. Although collagen, because of its unusual highly crystalline structure, is relatively inert to chemical and enzymic attack over the long periods of time, slow degradation can occur, and this can be ameliorated by artificial covalent cross-linking agents, for example, externally as leather and *in vivo* as prosthesis.

The interaction of these agents may occur with the ε-amino group of lysine residues, the carboxyl groups of glutamic and aspartic acids, or with hydroxyl groups. These groups project radially from the rod-like molecules, which are held in parallel alignment in the collagen fibre, thereby ensuring intermolecular and interfibrillar cross-linking (Fig. 2). These cross-links prevent the molecules from sliding past each other under load and therefore increase the mechanical strength of the fibre but also dramatically affect the thermal and biological properties of the fibre. Clearly, the nature and stability of these different chemical bonds produces a significant difference in the particular properties of the cross-linked product. However, above a certain level further cross-linking could have no effect in increasing the mechanical strength and the fibre would become brittle.

The increase in denaturation temperature is less obvious. The enzymically cross-linked mammalian native fibre shrinks to one-third to one-fourth of its original length when heated to about 65°C. However, chemical cross-linking can increase the shrinkage temperature to over 100°C depending on the nature of the cross-linking agent. The cross-links are formed between amino and carboxy side-chains of the triple helical regions of the molecule and might be expected to stabilise the molecules against thermal shrinkage but shrinkage occurs to the same extent. Shrinkage is due to the rupture of the hydrogen bonds as shown by the virtual constancy of the enthalpy whatever the increase in shrinkage temperature. Covington has proposed that the cross-links restrict the degrees of freedom of the α-chains thus reducing the rate of denaturation and

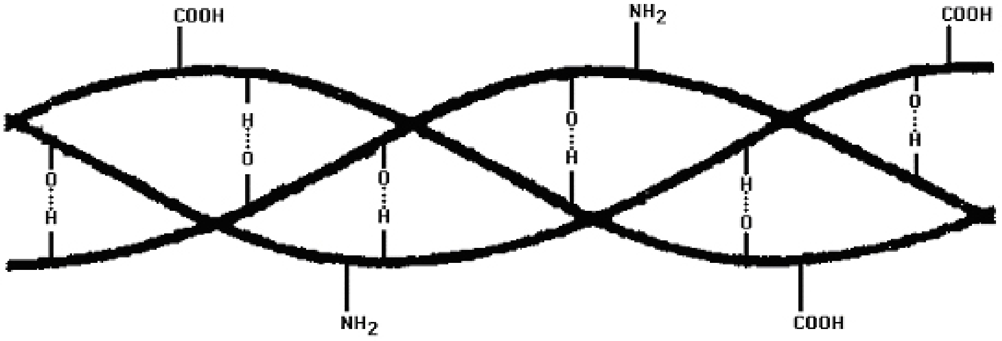

**FIGURE 1.** Collagen triple helix showing internal hydrogen bonding and radial side-chains.

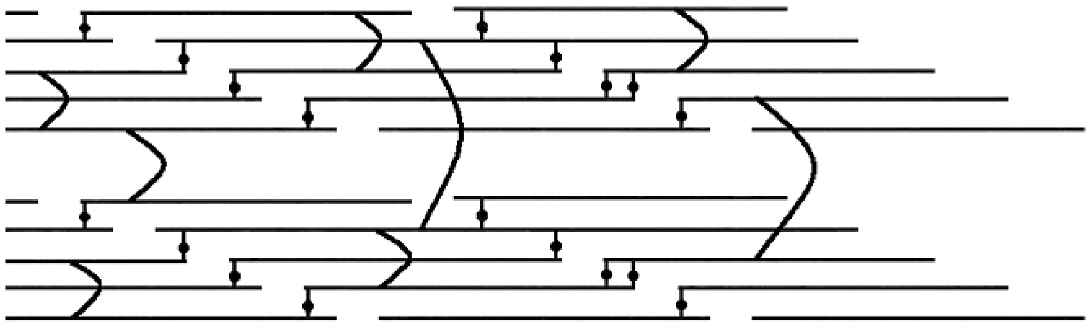

**FIGURE 2.** Quarter-stagger and end-overlap alignment of collagen molecules in the fibre, and typical positions of artificial chemical cross-links within and between microfibrils. Artificial cross-links: >; Natural cross-links: !

thereby raising the denaturation temperature[5]. However, we have previously shown a relationship between water-content and denaturation temperature and propose that the latter is increased on cross-linking by removal of water, (Miles and Bailey unpublished). The actual thermal stability of the artificial intermolecular cross-link may not be an important factor. Interestingly with stable cross-links there is a complete reversal of the structure on cooling, even to the extent of a reappearance of the 64-nm repeat axial periodicity. This cycle of shrinkage and reversal can be repeated and is referred to as the Ewald reaction.

Cross-linking also renders collagen fibres less susceptible to enzymic attack. The normal *in vivo* mode of collagen degradation involves mammalian collagenase, which attacks all three chains of the triple helix at a single point in the molecule, and by cathepsin which attacks the N- and C- terminals. These peptide cleavages result in triple helical fragments that denature at physiological temperatures, which are then degraded further by many enzymes, principally the gelatinases[6]. *In vitro* damage is often caused by bacterial collagenase, which has the ability to cleave the triple helix at numerous points along its length, thus slowly solubilising the fibre. The introduction of chemical cross-links between the helices prevents solubilisation and denaturation of the collagen by the above enzymes. The susceptibility to enzymes is further reduced by the dehydration of the fibres induced by the chemical cross-linking. The many different cross-linking reagents available can provide a very wide range of thermal and enzyme stabilities, or with a single reagent conditions can be varied to achieve a particular stability.

Although the majority of publications dealing with the chemistry of cross-linking of collagen refer to animal hides and the production of leather, the techniques apply to many other products. The cross-linking of short collagen-like sequences synthesised to mimic (and therefore termed 'biomimetics') specific properties of collagen is likely to become an important field in the near future as these peptides are now readily produced as recombinant peptides with known sequences.

### Mechanical and Thermal Characteristics of Collagen

All collagenous tissues have a mechanical function and therefore a requirement for high tensile strength. The alignment of the molecules in the fibre and the stabilisation in this position by the intermolecular cross-links endows collagen with a high degree of mechanical strength. The fibres are virtually inextensible, only elongating about 3% under load. The loads to break collagen are comparable with wood or steel wire.

The classical mechanical experiment determines stress and strain, that is, the load per unit cross-sectional area vs. the extent of the deformation in length of the fibre. The stress–strain curve is generally divided into three regions: the toe region, the linear region, and the plastic or rupture region. The toe region corresponds to the fibres becoming aligned and the crimp straightening out as the stress increases. In the linear region, the collagen fibres themselves take the stress and obey Hooke's Law. In the third region, relaxation occurs following slow rupture of the fibre. With increasing age of the animal, the toe region shortens and the linear part of the curve becomes steeper indicating a stiffer fibre, and there is an increase in the ultimate breaking load[4] as shown in Fig. 3. Similar changes occur on chemical cross-linking.

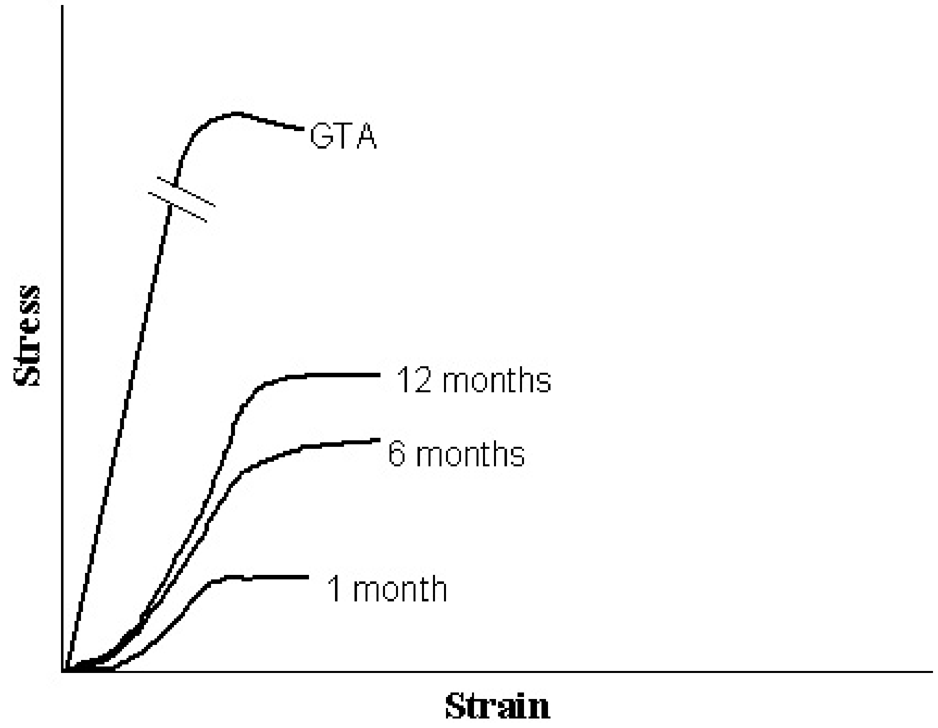

**FIGURE 3.** The changing stress-stain curve of immature and mature collagen fibres (tendon) and a typical chemically cross-linked (glutaraldehyde) fibre.

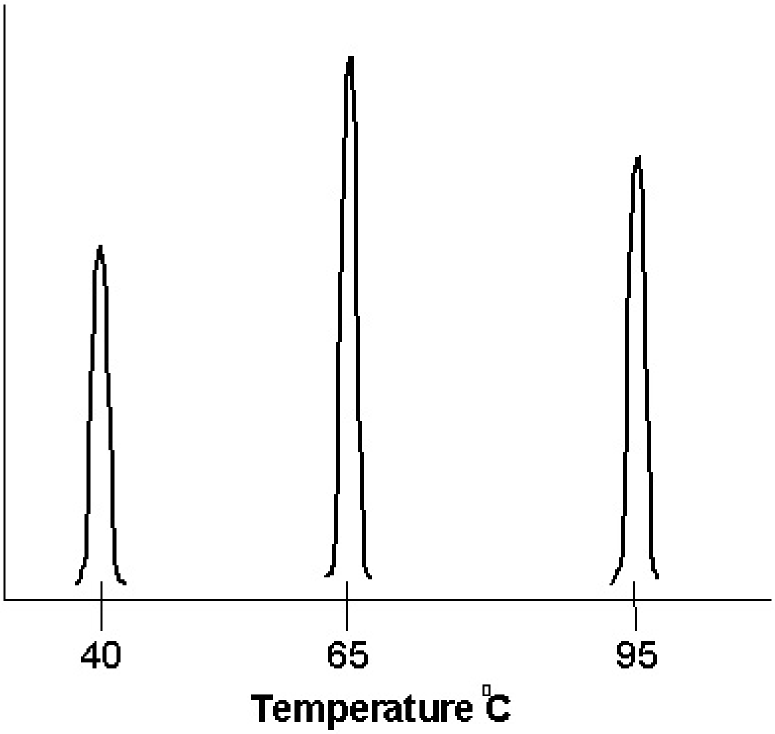

**FIGURE 4.** Thermograms of collagen in solution (tropocollagen), collagen aggregated in fibres, and a typical glutaraldehyde cross-linked fibre at 95 oC.

### *Thermal Properties*

The crystalline structure of the collagen molecule collapses to random chains on heating with a very sharp denaturation temperature. The rod-like molecules in solution denature at about 40°C for bovine and human skin, but can range from 15°C for cod skin to 50°C for Ascaris cuticle. This variation is correlated with the proportion of hydroxyproline in the particular specimen[7]. Denaturation of fibrous collagen occurs at 65°C, that is, an increase of about 27°C over the molecule in solution due to the energy of crystallization derived from the interactions between the molecules in the fibre.

The denaturation temperature of the collagen molecule in solution can be determined from the change in viscosity, visual observation of shrinkage, circular dichroism, or birefringence, but the most accurate method is differential scanning calorimetry (DSC)[8]. DSC has the great advantage of being able to determine both the denaturation of the collagen molecule and the collagen fibre (Fig. 4).

The enthalpy of denaturation, given by the area under the thermogram, is high for collagen, 70 J/g compared to 15 to 20 J/g for most other proteins, due to the crystalline nature of collagen. The stabilisation of the molecule is still controversial. Based on X-ray diffraction studies it was suggested that the stabilisation occurred through hydrogen-bonded water-bridges involving the hydroxy group of hydroxyproline[9] and supported by recent studies on the location of water molecules on synthetic collagen polypeptides[10]. There is certainly a reasonable correlation

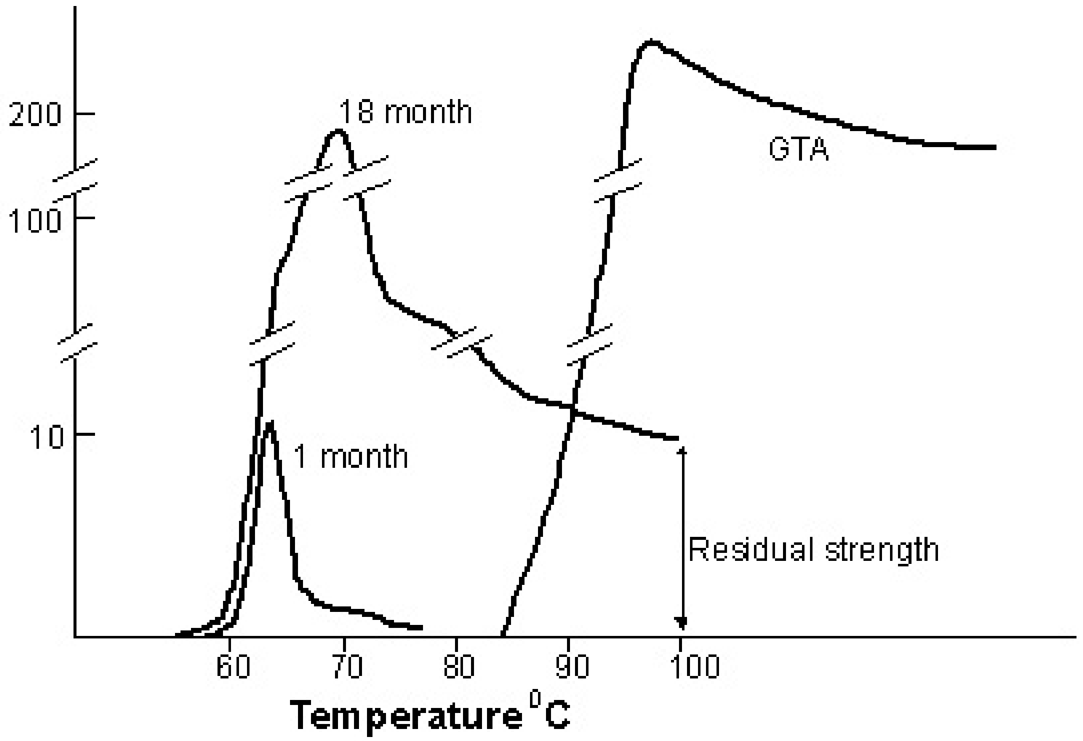

**FIGURE 5.** Hydrothermal isometric tension curves of immature and mature tendon and a typical (glutaraldehyde, GTA) cross-linked fibre.

between the hydroxyproline content and the denaturation temperature[7]. On the other hand Prockop and coworkers[11] suggested that hydroxyproline and water hydrogen bonding were not involved. This has recently been supported by Raines et al.[12] who proposed that stabilisation occurs through an inductive effect of the hydroxyl of hydroxyproline on the peptide backbone stabilising the trans configuration of the prolyl peptide bond.

Determination of the denaturation temperature and calculation of the enthalpy provides an indication of the integrity of the collagen molecule, degradation decreasing the enthalpy whilst cross-linking increases the denaturation temperature.

### Hydrothermal Isometric Tension

Collagen fibres shrink to about one quarter of their original length on heating above the denaturation temperature of about 65°C. However, if the fibre is held at constant length during heating the force generated in an attempt to shrink can be measured on a strain gauge.

The profile changes with increasing age, as more immature cross-links become thermally stable and there is a greater residual strength of the fibre, albeit only a fraction of that of the native fibre. Similar changes occur following chemical cross-linking (Fig. 5). Isometric tension therefore provides an estimate of the proportion of total and thermally stable cross-links.

### Resistance to Enzymic Degradation

Collagen in medical applications, e.g., as prostheses, require protection against tissue enzymic degradation. A common test for susceptibility to enzymes is to determine the amount of degradation *in vitro* due to bacterial collagenase. The collagen is added to a solution of collagenase and incubated at 37°C. The degradation can be followed by determining the hydroxyproline released into solution at regular intervals. As an example glutaraldehyde increases the degradation time tenfold.

#### *In Vivo Biocompatability*

The inherent antigenicity of collagen is very low but in some cases there is a mild inflammatory reaction to the collagen or to residual cross-link products. The most common test is to implant a collagen sponge under the skin of rats, and the sponge subjected to routine histological examination at regular intervals up to 21 days. The early presence of neutrophils is indicative of inflammatory response.

#### Uses of Collagen as a Biomimetic

The biocompatability of collagenous materials has resulted in their widespread use in medicine which has been reviewed previously[13,14,15,16]. They have been shown to promote wound healing as well as acting as haemostats, scaffolds to promote guided tissue regeneration, cell transplant devices, drug delivery systems, bioprostheses, and a range of ophthalmological devices. The vast array of tissues in which collagen plays a role in the human body is mirrored by the diversity of collagenous biomaterials produced to date. These materials differ markedly in their origin and the processing methods employed. In very simple terms these may be intact tissues such as heart valves[17] or reconstituted materials made from either soluble or insoluble collagen preparations. One common feature of all of these materials is the need for artificial cross-linking to control the residence time in the body, the mechanical properties, and to some extent the immunogenicity of the device. This has been achieved using a number of different types of cross-linking agents that react with specific amino acid residues on the collagen molecule imparting individual characteristics to the material formed.

### MECHANISMS OF COVALENT CROSS-LINKING

#### Reactions with ε-Amino Groups

Primary aldehydes react with the ε-amino groups of lysine residues in collagen and therefore readily form cross-links. Indeed, the reaction of all aldehydes as cross-linking agents is primarily with the ε-amino group of lysine and hydroxylysine and to a lesser extent guanidine, phenolic and thiol groups, virtually no reaction occurring with acid groups at slightly alkaline pH.

Although many aldehydes and dialdehydes have been employed, formaldehyde and glutaraldehyde are the major ones used commercially. These two compounds cross-link by different reactions.

#### *Formaldehyde*

The reactions with formaldehyde, generally under mild conditions and in dilute solution, are reversible, simple washing or steam distillation results in quantitative recovery of the formaldehyde.

$$\text{Coll}\rightarrow NH_2 + HCHO \rightarrow \text{Coll-NH-CHOH} \rightarrow \text{Coll NH–}CH_2\text{-NH-Coll}$$

Formaldehyde cross-linking is relatively inefficient, primarily because on storage it is converted to the less reactive paraformaldehyde, OH-$CH_2(CHOH)_2$ -CHO.

### *Glutaraldehyde*

Glutaraldehyde is the commonest aldehyde cross-linking agent in use for the preparation of collagenous biomaterials. Glutaraldehyde requires distillation prior to use in order to obtain the monomer for chemical studies since it readily forms condensation products such as aldols and polymeric complexes and eventually cyclic complexes on storage. Industrially it is generally used in equilibrium with the polymers.

In contrast to formaldehyde, glutaraldehyde forms chemically stable bonds. However, the nature of the cross-links is complex and they have not been fully characterised. Early studies suggested that the reactive species is a condensation polymer of glutaraldehyde, but Rydon and others[18] have demonstrated that glutaraldehyde is probably the reactive species although it is in equilibrium with several other reactive species. The aldehyde reacts with the ε-amino group of lysine, then condenses to form a heterocyclic compound which subsequently undergoes oxidation to a pyridinium ring (Fig. 6).

Glutaraldehyde increases the shrinkage temperature to over 80°C but the relative thermal stability of the whole homologous series of dialdehydes does not appear to have been extensively studied. It is not yet clear why glutaraldehyde forms such stable cross-links whilst succinaldehyde cross-links are unstable to hydrolysis and adipaldehyde cross-links collagen to only a limited extent. Possibly the addition of a single methylene group in the latter structure may lead to less stable heterocyclic ring compounds. Malondialdehyde, like glutaraldehyde, does not form the expected unstable double Schiff base, but a cyclic pyridine aldehyde[19].

The method by which cross-linking is achieved also has important effects upon the physical properties of the material formed. Duncan et al.[20] compared the glutaraldehyde cross-linking of aortic valves fixed under static or dynamic conditions. Excessive pressures or pulsation rates did not result in detectable differences between static and dynamically fixed tissues. However, at low pressure (<4.0 mmHg) and at vibration rates comparable to those observed *in vivo,* distinct differences in the viscoelastic properties of the valves were observed, i.e., there was an increase in the relaxation rates of dynamically fixed tissue to levels similar to fresh tissue. The authors suggest that dynamic fixation of tissues would retain the natural stress reducing mechanism of fresh tissue so improving their resistance to long-term mechanical fatigue. Dynamic fixation also caused a significant increase in shrinkage temperature of the valves compared to static fixation (89°C compared to 83°C).

Cross-linking using glutaraldehyde has also been shown to have marked effects upon the diffusion characteristics of collagen films[21]. This is of importance when the possible role of collagen as a carrier in drug delivery systems is considered.

Although widely used to stabilise collagen prostheses for application *in vivo,* the use of glutaraldehyde has been questioned due to its propensity to induce calcification[22] and cytotoxic effects probably due to the release of glutaraldehyde monomer[23]. Consequently, a variety of other cross-linking agents for use in medical applications are being investigated as possible replacements for glutaraldehyde.

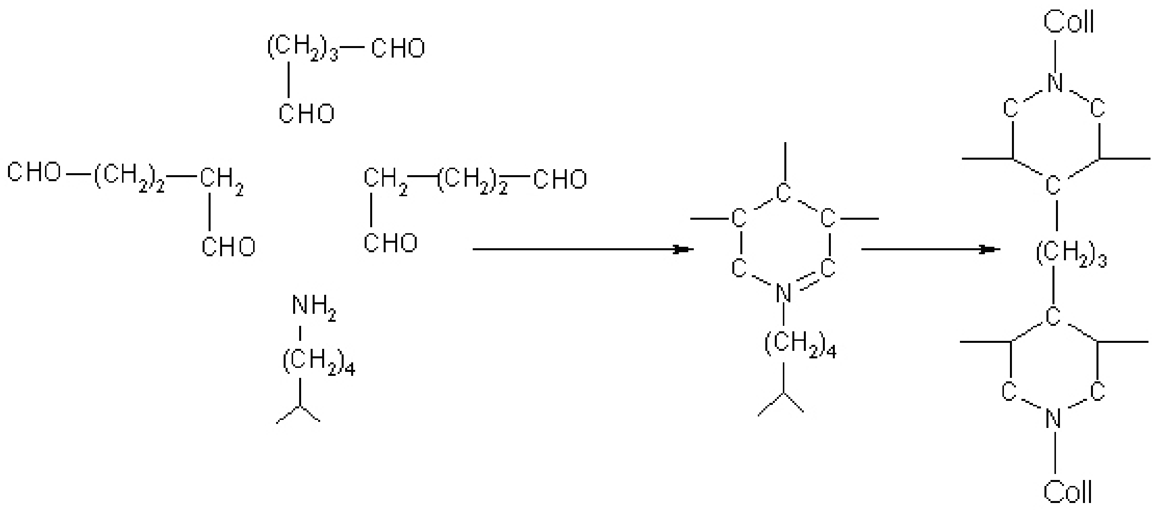

**FIGURE 6.** Reactions of glutaraldehyde with the ε-amino groups of collagen to form stable cyclic pyridinium ring cross-links.

### Isocyanates

Isocyanate reacts rapidly with ε–amino groups, hence di-isocyanates can be used as cross-linking agents. They react at room temperature and neutral pH. Studies have shown a decrease in solubility, and increase in both tensile strength and isometric tension generated on heating, all indicating considerable covalent cross-linking. A di-isocyanate could react with two amino groups to form an intermolecular cross-link.

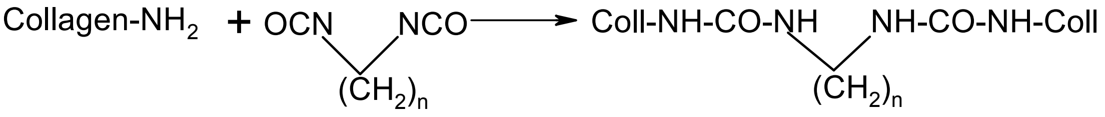

Hexamethylene di-isocyanate (HMIDC) has been used successfully[24] to cross-link dermal sheep collagen raising the shrinkage temperature of noncross-linked dermal tissue from 54 to 74°C. Such cross-linking increased the elongation to break from 141 to 163% and decreased the high-strain modulus from 26 to 16 MPa but did not affect the tensile strength of the tissue. HMDIC is only sparingly soluble in water and requires the use of surfactants during the cross-linking reaction to improve penetration.

Isothiocyanates react much more slowly than isocyanate, and generally the pH needs to be raised. A heterobifunctional reagent possessing both these groups could be employed to control the reaction.

### Quinones

Quinones or quininoid complexes react readily with the ε-amino lysine group of collagen.

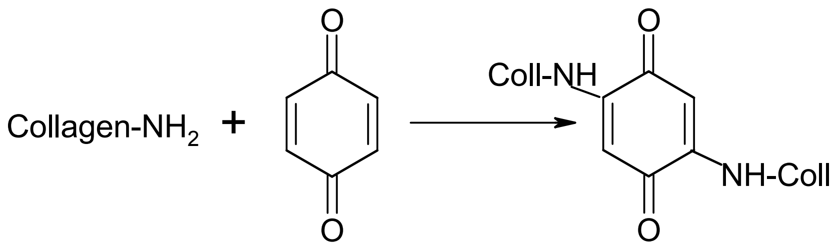

Stabilisation of some natural biological matrices can also be brought about by quinones *in vivo*, one example being the mechanism by which the collagenous byssus threads of mussels are modified to facilitate adhesion to the substratum[25,26]. In this reaction, *ortho*-catechols are enzymatically oxidised to *o*-quinones which polymerise the matrix. Koob and Hernandez[27] have exploited this phenomenon using the dicatechol nordihydroguaretic acid (NDGA) to stabilise reconstituted collagen giving fibres that have tensile properties greater than native tendons or fibres cross-linked with glutaraldehyde or carbodiimide.

Although, *o*-quinones can form covalent linkages with primary amines, Koob et al.[28] postulate that the stabilisation of the matrix is through the formation of an NDGA polymer in which the collagen fibrils are embedded rather than cross-linking between the protein and quinone. The same group has also shown that NDGA cross-linked fibres, with appropriate washing, have good biocompatability properties[28,29] and show great promise for prosthetic tendons.

### Reaction with Carboxyl Groups

The targeting of carboxy groups as the loci of intermolecular cross-links has gained increased attention in recent years. The most promising methods rely upon the activation of carboxyl groups on the polypeptide chain that can then react with amine groups on other chains.

Such methods can be split into two broad groups: carbodiimide method[30] (cyanamide is a simple carbodiimide NH=C=NH) and the acyl azide method[31]. These agents have the advantage of being able to induce the formation of very short intermolecular cross-links without themselves being incorporated into the final linkage.

$$\text{Coll-COOH} + 2 \times \text{R-NC=N-R'} \rightarrow \text{Coll-CO-NH-CO-Coll}$$

The most commonly used species in the first group is ethyl-3(3dimethylamino)propyl carbodiimide (EDC) which has been used successfully by a number of groups to cross-link collagenous tissues without inducing the side effects associated with glutaraldehyde. EDC reacts with the carboxy groups initially to form O-acylisourea groups, which then combine with ε-amino groups to form amide bonds. The extent of cross-linking and rate of cross-linking with EDC can be enhanced by the addition of N-hydroxysuccimide (NHS) to the cross-linking solution[32]. In this study the addition of NHS raised the shrinkage temperature of EDC cross-linked dermal collagen from 72 to 82°C. The combination of EDC and NHS also had significant effects upon the physical properties of the material and caused a decrease in the tensile strength, high strain modulus and an increase in elongation to break.

Amide bond formation similar to that induced by carbodiimides can also be affected by the use of the acyl azide method. This can be brought about by the methylation of carboxy groups to hydrazines followed by reaction with sodium nitride to form acyl azides or modified directly with agents such as diphenylphosphoryl azide (DPPA). The azides produced then react with amino groups to form amide cross-links. The reaction of collagen with 0.5% DPPA has been shown by Herbage and colleagues to give a level of cross-linking comparable to that of glutaraldehyde treatment but with much improved cytocompatability[33].

### Epoxides

Cyclic ethers are reactive due to the strained three-membered ether ring that can be opened by the nucelophilic attack of bases and acids. One such epoxide, epichlorhydrin, a key component of epoxy-glues, can form cross-links between carboxyl groups and amino groups.

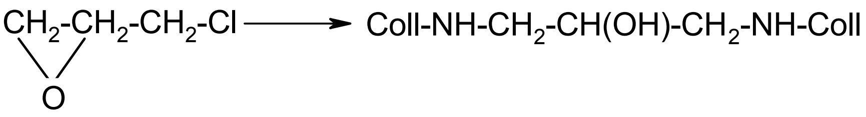

In the last 20 years the use of poly-epoxy compounds as biomaterial cross-linkers has also received increased attention, e.g., glycerol diglycidyl ether[34] and has been reviewed by Khor[17]. More recently the polyepoxide 1,4-butanediol diglycidyl ether (BDDGE) has been used to cross-link dermal sheep collagen at either alkaline or acidic pH. At acid pH cross-linking is predominantly via reaction with carboxylic acid groups whilst alkaline pH results in cross-links involving amine groups[35]. The pH at which the reaction takes place and hence the type of cross-link formed has a dramatic effect upon the physical properties of the material and biodegradation rates[36,37]. Dermal sheep collagen cross-linked at pH 4.5 using BDDGE had higher tensile strength and elongation at break than that cross-linked at pH 9.0 and was more flexible and pliable. However, resistance to biodegradation in the materials cross-linked at pH 4.5 was much poorer than those cross-linked at pH 9.0. Subsequent studies using EDC in a second cross-linking step improved the resistance of the acid BDDGE material whilst retaining its desirable physical properties[38].

### Enzyme-Generated Cross-Links

Transglutaminase (TGc) can be used to generate cross-links between glutamic acid and lysine. The isopeptide cross-link formed is common in nature, e.g., fibrin and keratin, but not in native collagen, although the extracted enzyme can be used *in vitro* to cross-link collagen. These crosslinks are also important in the assembly and remodelling of extracellular matrices[39]. The isopeptide bond is susceptible to acid and consequently isolation of the cross-link requires complete proteinase digestion.

$$\text{Coll-COOH} + \text{Coll-NH}_2 \longrightarrow \text{TG}_c \longrightarrow \text{Coll-CO-NH-Coll}$$

ε-(γ-glutamyl) lysine (collagen)

### Reversible Cross-Links

The formation of cross-links, which subsequently can be readily reversed under mild conditions, is of considerable value in identifying protein chain proximities or the nature of the cross-linking sites. The technique was established to study the topology of complex ribosomal subunit structures[40] and histone-histone interactions[41] reversing the cross-link with mild reduction of the disulphide bond. More sophisticated reagents are now employed, such as dimethylsuberimidate[42] and dimethyl 3 3 ‘ dithiobiscarboxylic acid bis(dimethylamide) which is readily reversed with dithiothreitol[43].

### Carbohydrate-Mediated Cross-Links

Nonenzymic glycosylation (Glycation) of collagen initially results in the formation of hexosyl lysine derivatives which are spontaneously stabilised by undergoing the Amadori rearrangement. This reactive species may then undergo further reaction to form stable covalent cross-links or degradation to glyoxal which, as a dialdehyde, readily forms cross-links with collagen. A complex heterocyclic product has been isolated from glycated collagen, termed pentosidine, and is derived from reaction of ribose with lysine and arginine[44] Other sugars, ribose and fructose react much faster than glucose. Glycation of collagen *in vivo* results in the most serious late complications of ageing and diabetes mellitus, causing dysfunction of retinal capillaries, renal tissues, and the cardiovascular system[45]. Initially glucose affects the collagen side-chains, which affects the cell-collagen interactions through the integrins and collagen-matrix component interactions[46], but the most damaging effects are caused by the formation of intermolecular cross-links which decrease the flexibility and permeability and inhibit turnover of tissues. We are not aware of any application of glucose as a cross-linking agent.

### Physical Treatments

#### *Radiation*

**X-ray or γ-irradiation** of collagen can result in two competing reactions: degradation through direct peptide bond cleavage which is predominant in the dry state, whilst covalent intermolecular cross-linking is predominant when irradiated wet. The nature of the cross-links has not been determined, but the formation of peroxy radicals indicated the formation of diaromatic or disulphide residues.

Degradation in the dry state occurs through direct action on the peptide bonds increasing the solubility and reducing the tensile strength. In contrast, irradiation in the wet state decreases the solubility, the tensile strength slightly but a significant increase in thermal isometric tension due to the formation of thermally stable cross-linking initiated by free radical formed in the water[47].

#### *UV Light*

UV irradiation of wet collagen fibres causes rapid insolubility. The mechanism is presumably similar to γ-irradiation since the major effect in both cases is the formation of the hydroxyl radical ($OH^{\bullet}$) from water. The OH radical attacks the peptide backbone to produce peptide radicals (-NH-$C^{\bullet}$-CO-) which can interact to form a cross-link. We have shown that rapid degradation of phenylalanine and tyrosine can occur but the nature of the cross-link remains to be elucidated.

Previous studies have suggested that dityrosine is formed as a cross-link but we failed to detect the presence of this compound[48].

#### Dye-Mediated Photo-Oxidation

This method has been used to produce bovine and porcine pericardial tissue that was resistant to calcification, had low immunogenicity, and was biocompatible[49,50]. The physical and biological properties of tissue treated in this way are consistent with the formation of intermolecular cross-links, i.e., the presence of high molecular weight aggregates, resistance to cyanogen bromide digestion, and proteolyic cleavage although the identity of the cross-linking species is not known[51]. However, the partial reversal of cyanogen bromide susceptibility by reduction indicates that methionine residues may be involved[51]. More recent work has also indicated that histidine residues are extensively modified by this reaction[52]. One interesting aspect of this method is the fact that it does not result in a significant increase in the shrinkage temperature[53]. Thermal stability as measured by thermal relaxation analysis is, however, improved by this treatment.

#### Dehydration

Extensive dehydration, particularly under high vacuum is well known to cause insolubility either through hydrophobic or covalent bonds. Yannas and Tobolski[54] proposed that a covalent amide bond was formed between the carboxyl and amino side-chains. Their proposal was primarily based on the fact that acetylation of the amino group and esterification of the carboxyl group prevented the insolubilisation and a reduction in amino and carboxy groups during drying. Direct confirmation by isolation of the cross-links employing enzyme digestion has not been reported. Interestingly drying produces the highest shrinkage temperature, with the triple helix clearly maintaining its integrity.

### Chemical Modification of Collagen

**Reduction —** The major intermolecular covalent cross-links in the tissues of immature animals formed through lysyl oxidase as aldimine and keto-imines are acid and heat labile. Mild reduction with sodium borohydride stabilises these cross-links rendering the collagen insoluble, increasing the mechanical strength and the denaturation temperature[4,55].

**Oxidation —** The stability of collagen can be increased by mild oxidation employing potassium persulphate, or better, chloramine-T.

**Alkaline Treatment** — Results in the decomposition of serine, cysteine, and threonine by the elimination of water and hydrogen sulphide to form lysinoalanine as a cross-link[56]. The formation of lysinoalanine in food processing can have both nutritional and toxicological effects. The formation can take place by β-elimination followed by addition or substitution.

- **β-Elimination**

$$\left.\begin{array}{c} {}_{\alpha}\text{C-CH}_2\text{-OH} \\ \text{Serine} \\ {}_{a}\text{C-CH}_2\text{-SH} \\ \text{Cysteine} \end{array}\right\} \longrightarrow \underset{\text{dehydroalanine}}{{}_{a}\text{C=CH}_2} + \text{lysine} \longrightarrow \underset{\text{lysinoalanine}}{{}_{a}\text{C-CH}_2\text{-NH-(CH)}_4\text{-C}_a}$$

- **Substitution**

$$\underset{\text{Serine}}{{}_{\alpha}\text{C-CH}_2\text{-OH}} + \text{lysine} \longrightarrow \underset{\text{lysinoalanine}}{{}_{a}\text{C-CH}_2\text{-NH-(CH)}_4\text{-C}_a}$$

### SUMMARY

The ability to modify collagen, and collagen-like polypeptides, processed as films, tubes, gels, etc., to the required mechanical strength, melting temperature, and biocompatability can be achieved by a variety of cross-linking agents. All these parameters can be met by correct choice of cross-link as shown in Tables 1 and 2.

The cross-linking of purified native collagen fibres and recombinant collagen polypeptides is crucial to controlling their residence time, being particularly necessary for their long-term function in the case of prostheses, such as heart valves. Cross-linking achieves a considerable increase in the resistance to enzymic degradation. At the same time, choice of the correct cross-link is necessary to avoid an antigenic reaction against the collagen or biocompatability of the cross-link itself or its degradation products. Herbage and colleagues[33,57] have surveyed a number of cross-linking agents and reported that azides (DPP) and carbodiimides (EDC) both produce physical characteristics equivalent to glutaraldehyde but without any signs of biocompatability problems.

The increase in mechanical strength is due to the cross-linking between the fibres preventing the fibres sliding past each other, and the enzyme retention time due to the increased inability of the enzyme to access the molecules. The increase in denaturation temperature may be due to increased rigidity imposed on the triple helix ensuring a slow rate of denaturation and hence a higher melting point as proposed by Covington[5]. However, the increase is highest for the dried fibre demonstrating the importance of water in the determination of the melting point. Collagen based biomaterials are used in a wide variety of applications but the second generation involves the use of recombinant sequences of collagen chosen for their preferred properties and should be even more effective biomimetics.

**TABLE 1**
**Effect of Covalent Cross-Linking on the Mechanical Properties (Adapted from Table 2 Koob et al.[27])**

| | Tensile strength (MPa) | Modulus (MPa) | Failure strain (%) | Reference |
|---|---|---|---|---|
| NDGA | 91 | 582 | 10 | [27] |
| Glutaraldehyde | 61 | 489 | 8 | [27] |
| | 64 | 456 | 15 | [58] |
| | 31 | 190 | 22 | [59] |
| | 55 | — | — | [60] |
| Carbodiimide | 12 | 45 | 37 | [59] |
| | 27 | 180 | 18 | [58] |
| Tendon | | | | |
| Bovine digital ext. | 47 | — | — | [27] |
| Rabbit digital ext. | 43 | 366 | 15 | [27] |
| Rat tail | 39 | 570 | 8 | [58] |

**TABLE 2**
**Comparative Effects of Covalent Cross-Linking on Shrinkage Temperature**

| Type of reagent | Approx. Temp. | Reacting Group | References |
|---|---|---|---|
| **Aldehydes** | | | |
| Formaldehyde | +15°C | ε-Amino of lysine | [5] |
| Malondialdehyde | +20°C | ε -Amino of lysine | [19] |
| Glutaraldehyde | +30°C | ε -Amino of lysine | [20] |
| **Phosphorylazide** | +20°C | ε -Amino of lysine | [30] |
| **Di-isocyanate** | +20°C | ε -Amino of lysine | [32] |
| **Glycation** | +10°C | Amino/guanidine | [44,45] |
| **Carbodiimides** | +10°C | Carboxy group | [30,33] |
| **Transglutaminase** | ? | Carboxyl group | [39] |
| **Poly-epoxy (BDDGE)** | | | |
| (at pH 9.0) | +5°C | Carboxy/amino | [17,38] |
| (at pH 4.5) | +1-2°C | Carboxy/amino | [17,38] |
| **Dehydration** | Approx. 120°C | Carboxy/amino | [54] |
| **Reduction ($BH_4$)** | +5°C | Schiff base | [55] |

## References

1. Kielty, C.M., Hopkinson, I., and Grant, M.E. (1993) The collagen family: structure, assembly and organisation in the extracellular matrix. In *Connective Tissue and Its Heritable Disorders*. Royce, P.M. and Steinman, B., Eds. Wiley-Liss, New York. p. 103–148.
2. Comper, W.D. (1996) *Extracellular Matrix*. Harwood Academic Publishing, Australia.
3. Bailey, A.J. and Paul, R.G. (1998) Collagen: a not so simple protein. *J. Soc. Leather Technol. Chem.* **82**, 104–110.
4. Bailey, A.J., Paul, R.G., and Knott, L. (1998) Mechanisms of maturation and ageing of collagen. *Mech. Ageing Dev.* **106**, 1–56.
5. Covington, A.D. (1997) Modern tanning chemistry. *Chem. Soc. Rev.* **26**, 111–126.
6. Bailey, A.J. (2000) The fate of collagen implants in tissue defects. *Wound Repair Regen*. **8,** 5–12.
7. Burjanadze, T.V. (1982) Evidence for the role of 4-hydroxyproline localized in the third position of the triplet (gly-x-y) in adaptational changes in the thermostability of a collagen molecule and collagen fibrils. *Biopolymers* **21**, 1489–1501.
8. Miles, C.A., Burjanadze, T.V., and Bailey, A.J. (1995) The kinetics of the thermal denaturation of collagen in unrestrained rat tail tendon determined by differential scanning calorimetry. *J. Mol. Biol.* **245**, 437–446.
9. Ramachandran, G.N., Bansal, M., and Bhatnagar, R.S. (1973) A hypothesis on the role of hydroxyproline in stabilising collagen structure. *Biochim. Biophys. Acta* **323**, 166–171.
10. Bella, J., Brodsky, B., and Berman, H.M. (1995) Hydration structure of a collagen peptide. *Structure* **3**, 893–906.
11. Engel, J., Chen, H.-T., and Prockop, D.J. (1977) The triple helix-coil conversion of collagen-like polytripeptides in aqueous and non-aqueous solvents. *Biopolymers* **16**, 601–622.
12. Holmgren, S.K., Taylor, K.M., Bretscher, L.E., and Raines, R.T. (1998) Code for collagen's stability deciphered. *Nature* **392**, 666–667.
13. Ramshaw, J.A.M., Werkmeister, J.A., and Glattauer, V. (1995) Collagen-based biomaterials. *Biotechnol. Genet. Eng. Rev.* **13**, 335–382.
14. Pachence, J.M. (1996) Collagen-based devices for soft tissue repair. *J. Biomed. Mater. Res.* **33**, 35–40.
15. Friess, W. (1998) Collagen - biomaterial for drug delivery. *Eur. J. Pharm. Biopharm.* **45**, 113–136.
16. Lee, C.H., Singla, A., and Lee, Y. (2001) Biomedical applications of collagen. *Int. J. Pharm.* **221**, 1–22.
17. Khor, E. (1997) Methods for the treatment of collagenous tissues for bioprostheses. *Biomaterials* **18**, 95–105.
18. Hardy, P.M., Nicholls, A.C., and Rydon, H.N. (1976) The nature of the cross-linking of proteins by glutaraldehyde. 1. *J. Chem. Soc. Perkin Trans.* **1**, 958–961.
19. Slatter, D.A., Paul, R.G., Murray, M., and Bailey, A.J. (1999) Reactions of lipid-derived malondialdehyde with collagen. *J. Biol. Chem.* **274**, 19661–19669.
20. Duncan, A.C., Boughner, D., and Vesely, I. (1996) Dynamic glutaraldehyde fixation of a porcine aortic valve xenograft .1. Effect of fixation conditions on the final tissue viscoelastic properties. *Biomaterials* **17**, 1849–1856.
21. Ho, H.O., Lin, C.W., and Sheu, M.T. (2001) Diffusion characteristics of collagen film. *J. Controlled Release* **77**, 97–105.
22. Golomb, G., Schoen, F.J., Smith, M.S., Linden, J., Dixon, M., and Levy, R.J. (1987) The role of glutaraldehyde-induced crosslinks in calcification of bovine pericardium used in valve bioprosthesis. *Am. J. Pathol.* **127**, 122–130.
23. Wiebe, D., Megerman, J., L'Italien, G.J., and Abbott, W.M. (1988) Glutaraldehyde release from ascular prostheses of biological origin. *Surgery* **104**, 26–33.
24. Olde Damink, L.H.H., Dijkstra, P.J., Vanluyn, M.J.A., Vanwachem, P.B., Nieuwenhuis, P., and Feijen, J. (1995) Cross-linking of dermal sheep collagen using hexamethylene diisocyanate. *J. Mater. Sci. Mater. Med.* **6**, 429–434.
25. Brown, C.H. (1952) Some structural proteins of mytilus edulas. *Q. J. Microsc. Sci.* **93**, 487–502.
26. Waite, J.H., Housley, T.J., and Tanzer, M.L. (1985) Peptide repeats in mussel glue protein. *Biochemistry* **24**, 5010–5014.
27. Koob, T.J. and Hernandez, D.J. (2002) Material properties of polymerized NDGA-collagen composite fibers: development of biologically based tendon constructs. *Biomaterials* **23**, 203–212.
28. Koob, T.J., Willis, T.A., and Hernandez, D.J. (2001) Biocompatibility of NDGA-polymerized collagen fibers. I. Evaluation of cytotoxicity with tendon fibroblasts in vitro. *J. Biomed. Mater. Res.* **56,** 31–39.
29. Koob, T.J., Willis, T.A., Qiu, Y.S., and Hernandez, D.J. (2001) Biocompatibility of NDGA-polymerized collagen fibers. II. Attachment, proliferation, and migration of tendon fibroblasts in vitro. *J. Biomed. Mater. Res.* **56**, 40–48.
30. Weadock, K., Olson, R.M., and Silver, F.H. (1983) Evaluation of collagen crosslinking techniques. *Biomater. Med. Dev. Artif. Organs* **11**, 293–318.

31. Petite, H., Rault, I., Huc, A., Menasche, P., and Herbage, D. (1990) Use of the acyl azide method for cross-linking collagen-rich tissues such as pericardium. *J. Biomed. Mater. Res.* **24**, 179–187.
32. Olde Damink, L.H.H., Dijkstra, P.J., vanLuyn, M.J.A., vanWachem, P.B., Nieuwenhuis, P., and Feijen, J. (1996) Cross-linking of dermal sheep collagen using a water-soluble carbodiimide. *Biomaterials* **17**, 765–773.
33. Petite, H., Duval, L.L., Frei, V., Abdulmalak, N., Sigotluizard, M.F., and Herbage, D. (1995) Cytocompatibility of calf pericardium treated by glutaraldehyde and by the acyl azide methods in an organotypic culture model. *Biomaterials* **16**, 1003–1008.
34. Tu, R., Lu, C.L., Thyagarajan, K., Wang, E., Nguyen, H., Shen, S., Hata, C., and Quijano, R.C. (1993) Kinetic-study of collagen fixation with polyepoxy fixatives. *J. Biomed. Mater. Res.* **27**, 3–9.
35. Shecter, L., Wynstra, J., and Kurkjy, R.P. (1956) Glycidyl ether reactions with amines. *Ind. Eng. Chem.* **48**, 94–97.
36. van Wachem, P.B., Zeeman, R., Dijkstra, P.J., Feijen, J., Hendriks, M., Cahalan, P.T., and van Luyn, M.J.A. (1999) Characterization and biocompatibility of epoxy-crosslinked dermal sheep collagens. *J. Biomed. Mater. Res.* **47**, 270–277.
37. Zeeman, R., Dijkstra, P.J., van Wachem, P.B., van Luyn, M.J.A., Hendriks, M., Cahalan, P.T., and Feijen, J. (1999) Crosslinking and modification of dermal sheep collagen using 1,4-butanediol diglycidyl ether. *J. Biomed. Mater. Res.* **46**, 424–433.
38. Zeeman, R., Dijkstra, P.J., van Wachem, P.B., van Luyn, M.J.A., Hendriks, M., Cahalan, P.T., and Feijen, J. (1999) Successive epoxy and carbodiimide cross-linking of dermal sheep collagen. *Biomaterials* **20**, 921–931.
39. Aeschlimann, D. and Thomazy, V. (2000) Protein crosslinking in assembly and remodelling of extracellular matrices: the role of transglutaminases. *Connect. Tissue Res.* **41**, 1–27.
40. Somer, A. and Traut, R.R. (1976) Identification of neighbouring protein pairs in the Escherichia coli 30s ribosomal subunit by crosslinking with methyl-4-mercaptobutyrimidate. *J. Mol. Biol.* **106**, 995–1015.
41. Thomas, J.O. and Kornberg, R.D. (1975) Cleavable cross-links in the analysis of histone-histone associations. *FEBS Letts*. **58**, 353–358.
42. Norcum, M.T. and Warrington, J.A. (1998) Structural analysis of the multienzyme aminoacyl tRNA synthetase complex. A three-domain model based on reversible chemical cross-links. *Protein Struct*. **7**, 79–87.
43. Sharma, K.K. and Ortwerth, B. (1995) Effects of cross-linking the chaperone-like function of alpha-crystallin. *Exp. Eye Res*. **61**, 413–421.
44. Sell, D.R. and Monnier, V.M. (1989) Structure elucidation of a senescence cross-link from human extracellular matrix. Implication of pentoses in the aging process. *J. Biol. Chem.* **264**, 21597–21602.
45. Paul, R.G. and Bailey, A.J. (1996) Glycation of collagen: the basis of its central role in the late complications of ageing and diabetes. *Int. J. Biochem. Cell Biol.* **28**, 1297–1310.
46. Paul, R.G. and Bailey, A.J. (1999) The effect of advanced glycation end-product formation upon cell-matrix interactions. *Int. J. Biochem. Cell Biol.* **31**, 653–660.
47. Bailey, A.J. (1968) Effects of ionizing radiation on connective tissue components. *Int. Rev. Connect. Tissue Res.* **4**, 233–281.
48. Miles, C.A., Sionkowska, A., Hulin, S.L., Sims, T.J., Avery, N.C., and Bailey, A.J. (2000) Identification of an intermediate state in the helix-coil degradation of collagen by ultraviolet light. *J. Biol. Chem.* **275**, 33014–33020.
49. Moore, M.A. and Phillips, R.E. (1997) Biocompatibility and immunologic properties of pericardial tissue stabilized by dye-mediated photooxidation. *J. Heart Valve Dis.* **6**, 307–315.
50. Moore, M.A. and Adams, A.K. (2001) Calcification resistance, biostability, and low immunogenic potential of porcine heart valves modified by dye-mediated photooxidation. *J. Biomed. Mater. Res.* **56**, 24–30.
51. Moore, M.A., Bohachevsky, I.K., Cheung, D.T., Boyan, B.D., Chen, W.M., Bickers, R.R., and McIlroy, B.K. (1994) Stabilization of pericardial tissue by dye-mediated photooxidation. *J. Biomed. Mater. Res.* **28**, 611–618.
52. Adams, A.K., Talman, E.A., Campbell, L., McIlroy, B.K., and Moore, M.A. (2001) Crosslink formation in porcine valves stabilized by dye- mediated photooxidation. *J. Biomed. Mater. Res.* **57**, 582–587.
53. Moore, M.A., Chen, W.M., Phillips, R.E., Bohachevsky, I.K., and McIlroy, B.K. (1996) Shrinkage temperature versus protein extraction as a measure of stabilization of photooxidized tissue. *J. Biomed. Mater. Res.* **32**, 209–214.
54. Yannas, I.V. and Tobolski, A.V. (1967) Cross-linking of gelatin by dehydration. *Nature* **215**, 509–560.
55. Bailey, A.J. (1968) Intermediate labile intermolecular cross-links in collagen fibres. *Biochim. Biophys. Acta* **160**, 447–453.
56. Bohak, Z. (1968) $N^{\varepsilon}$( dl-2-amino-2-carboxyethyl)-l-lysine: a new amino acid formed on alkaline treatment of proteins. *J. Biol. Chem.* **239**, 2878.
57. Rault, I., Frei, V., Herbage, D., AbdulMalak, N., and Huc, A. (1996) Evaluation of different chemical methods for cross-linking collagen gel, films and sponges. *J. Mater. Sci. Mater. Med.* **7**, 215–221.

58. Kato, Y.P., Christiansen, D.L., Hahn, R.A., Shieh, S.J., Goldstein, J.D., and Silver, F.H. (1989) Mechanical-properties of collagen-fibers - a comparison of reconstituted and rat tail tendon fibers. *Biomaterials* **10**, 38–41.
59. Goldstein, J.D., Tria, A.J., Zawadsky, J.P., Kato, Y.P., Christiansen, D., and Silver, F.H. (1989) Development of a reconstituted collagen tendon prosthesis - a preliminary implantation study. *J. Bone Joint Surg. Am. Vol.* **71A**, 1183–1191.
60. Dunn, M.G., Avasarala, P.N., and Zawadsky, J.P. (1993) Optimization of extruded collagen-fibers for acl reconstruction. *J. Biomed. Mater. Res.* **27**, 1545–1552.
